# Development of the ‘Canteen Scan’: an online tool to monitor implementation of healthy canteen guidelines

**DOI:** 10.1186/s12889-018-5974-8

**Published:** 2018-09-10

**Authors:** I. J. Evenhuis, N. L. W. J. Wezenbeek, E. L. Vyth, L. Veldhuis, M. P. Poelman, D. Wolvers, J. C. Seidell, C. M. Renders

**Affiliations:** 1 Department of Health Sciences, Faculty of Science, Vrije Universiteit Amsterdam, Amsterdam Public Health research institute, De Boelelaan 1085, 1081 HV Amsterdam, the Netherlands; 2 Netherlands Nutrition Centre, PO Box 85700, 2508 CK The Hague, the Netherlands; 3 Department of Human Geography and Spatial Planning, Faculty of Geosciences, Utrecht University, Heidelberglaan 2, 3584 CS Utrecht, the Netherlands

**Keywords:** Digital assessment, Food, Canteens, Public setting, Environment, Nutrition policy, Content validity

## Abstract

**Background:**

To improve the availability and accessibility of healthier food and drinks in schools, sports and worksites canteens, national Guidelines for Healthier Canteens were developed by the Netherlands Nutrition Centre. Until now, no tool was available to monitor implementation of these guidelines. This study developed and assessed the content validity and usability of an online tool (the ‘Canteen Scan’) that provides insight into and directions for improvement of healthier food products in canteens.

**Methods:**

The Canteen Scan was developed using a three-step iterative process. First, preliminary measures and items to evaluate adherence to the guidelines were developed based on literature, and on discussions and pre-tests with end-users and experts from science, policy and practice. Second, content validity of a paper version of the Canteen Scan was assessed among five end-users. Third, the online Canteen Scan was pilot tested among end-users representing school canteens. Usability was measured by comprehensibility, user-friendliness, feasibility, time investment, and satisfaction.

**Results:**

The content validity of the Canteen Scan was ensured by reaching agreement between stakeholders representing science, policy and practice. The scan consists of five elements: 1) basic conditions (e.g. encouragement to drink water and availability of policy regarding the guidelines), 2) product availability offered on displays (counter, shelf) and 3) in vending machines, 4) product accessibility (e.g. promotion and placement of products), and 5) an overall score based on the former elements and tailored feedback for creating a healthier canteen. The scan automatically classifies products into healthier or less healthy products. Pilot tests indicated good usability of the tool, with mean scores of 4.0–4.6 (5-point Likert scale) on the concepts comprehensibility, user-friendliness and feasibility.

**Conclusion:**

The Canteen Scan provides insight into the extent to which canteens meet the Dutch Guidelines for Healthier Canteens. It also provides tailored feedback to support adjustments towards a healthier canteen and with the scan changes over time can be monitored. Pilot tests show this tool to be usable in practice.

## Background

Although average life expectancy has increased, in general people have more unhealthy life-years, particularly due to an increase in premature non-communicable diseases including cardiovascular diseases, diabetes and cancer [1–3]. An unhealthy diet is one of the drivers of this trend [4]. Dietary behaviour has shown an unfavourable change, influenced by factors on the individual level like behavioural determinants and demographic factors as well as factors within the food environment [5, 6]. Public food settings have tended to increase the offer (availability), placement and promotion (accessibility) of unhealthy calorie-dense food and beverages [7]. These changes encourage people to consume these foods and drinks more frequently [8–11]. It is important to change the unhealthy food environment into one that helps individuals to make healthier food choices [12].

In recent years, efforts have been made to create healthier food environments. Attention increased towards school food policy formulation, research on food environment measurements, and environmental interventions in settings as home, school and worksite [13–15]. Increasing the availability and/or accessibility of healthier products has proven to be effective in stimulating healthier food choices (e.g. by placing more fruit/vegetables on display, advertisement for vegetables, or reducing the number of less healthy products at the point of purchases) [12, 16–20]. Altering the environment to make the healthier option the easier, default option, without restricting the consumer’s freedom of choice, is also known as ‘nudging’ [21]. Nudges are cheap to perform and require minimal effort. Examples of effective nudging strategies are: to offer a variety of healthier products instead of just one (e.g. different types of fruits), to position healthier products more attractively along the shopping route, and to increase the convenience of healthier products (e.g. sliced fruit instead of a single piece) [22, 23]. Especially in public settings, like school/sports canteens and worksite cafeterias, where people spend much time and may consume a significant amount of their daily caloric intake, nudging has received consumers’ approval and has the potential to positively affect customers’ dietary behaviour [11, 24, 25]. Moreover, visitors address the need for a larger range of healthy products [26] and schools, sports associations and companies have become increasingly interested in offering a healthier canteen by making use of nudges [27, 28].

The Dutch Ministry of Health, Welfare and Sport has set a policy target to increase the number of schools with a healthier canteen [29, 30]. Due to the absence of international consensus on how to define a ‘healthy canteen’ [31], the ‘Guidelines for Healthier Canteens’ were developed by the Netherlands Nutrition Centre in collaboration with experts in the field of nutrition and health behaviour. These guidelines are based on Dutch nutritional guidelines, experiences with the Dutch school canteen program, and general research on influencing food choices [32–34]. The Guidelines for Healthier Canteens aim to change the food environment in school/sports canteens and worksite cafeterias by improving the availability and accessibility of healthier foods. Availability is defined as the presence of products that can be bought. Accessibility is defined as product promotion and placement [33]. The next step is to implement these guidelines throughout the Netherlands. This requires effective infrastructure and support [35–37]. Therefore, we aimed to develop a user-friendly online tool that i) helps stakeholders to understand and implement the guidelines, ii) facilitates monitoring of the canteen’s status and changes over time regarding availability and accessibility of food/beverages, and iii) that provides tailored feedback and advises how to make the canteen healthier [13, 38]. In addition to the Netherlands, also in several other countries efforts have been made to create school food policies, such as guidelines, procedures or rules to enable a healthier school food environment [36, 39]. However, often the actual implementation of these policies can be improved and surveillance is recommended to monitor implementation over time [35, 36, 40]. Therefore, tools to monitor the implementation of these policies are required [35, 39, 41, 42].

Various measurement tools are available to assess product availability/accessibility in the consumer food environment [15, 31, 43, 44]. For example, in the United States the Nutrition Environment Measurement Survey for Stores (NEMS-S) and Restaurants (NEMS-R) are regularly used to assess the food environment and have also been tested on reliability and validity [45, 46]. The NEMS started as a tool to assess the availability, price and quality of products in stores, and to assess the availability, facilitators, barriers, pricing and signage/promotion in restaurants. Meanwhile, a version for vending machines is also available [47]. Unfortunately, none of the available tools were suitable to monitor Dutch canteens due to differences in nutritional guidelines and definitions of accessibility [15, 44]. Also, Dutch canteens differ from other countries regarding the products sold because in the Netherlands, most children bring their lunch from home, so in school canteens snacks are the main purchase. Moreover, the psychometric properties of these instruments have not always been properly evaluated [15, 44].

One of the first properties that should be assessed is the degree to which the content of the instrument is an adequate reflection of the construct to be measured (content validity) [48]. In addition, to facilitate the use of the tool by different stakeholders and to ensure clear and usable feedback is provided by the tool, it is recommended to develop it in a close collaboration between science and practice [49, 50]. Therefore, this paper describes the development (in close collaboration between practice and research) and assessment of the content validity and usability of the ‘Canteen Scan’.

## Methods

### Guidelines for Healthier Canteens as a conceptual framework

**Fig. 1:**
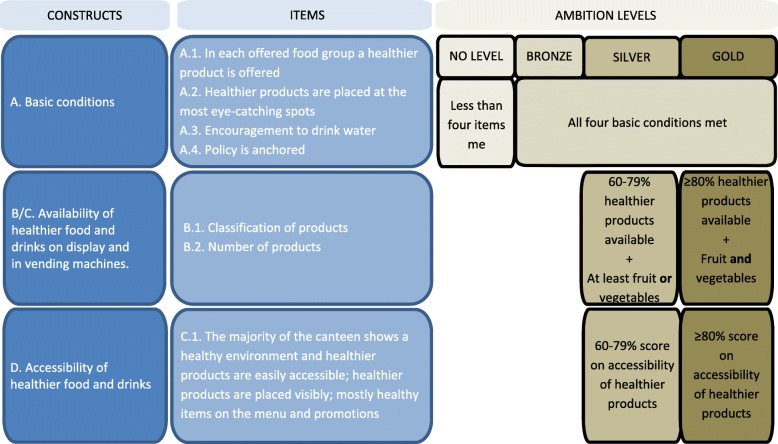
Conceptual framework for the Canteen Scan based on the Guidelines for Healthier Canteens

The Guidelines for Healthier Canteens consist of three predefined ambition levels bronze, silver, gold; these correspond to an increasingly healthy range of foods and drinks being available and accessible [33]. The levels are awarded based on four constructs: A) a set of basic conditions. This is a mix of availability, accessibility and policy items, all of which need to be present in a healthier canteen. B/C) the percentage of healthier products on display and in vending machine, i.e. healthier products that are available in the total range of products. D) a score on the accessibility of healthier products (see Fig. 1). Healthier and less healthy products are classified according to the Dutch Food-Based Dietary guidelines, based on five food groups known as the Wheel of Five [34]. In the Guidelines for Healthier Canteens, healthier products are defined as foods that are included in the Wheel of Five such as whole wheat bread, fruits and vegetables, semi-skimmed milk, and low fat cheese, and small portions of less healthy foods with limited calories, saturated and trans-fat, sodium and added sugar [33, 34]. These four constructs formed the conceptual framework of the tool to be developed. Further, as no additional criteria to assess the four constructs were defined in the guidelines, further operationalisation was necessary to measure adherence to the guidelines.

### Study design and setting

The study was conducted between December 2014 and January 2016. We used a 3-step iterative process of drafting, and continuous evaluation and revision. This design was based on recommendations for developing and evaluating measurement instruments [48–51]. They emphasize to develop a measurement instrument in an iterative process based on a clear definition of the construct to be measured, with people who have expertise in the field and to keep the practical application in mind [49–51]. The tool was therefore developed in multiple cycles of development, evaluation and adaptations and each cycle was properly evaluated based on input of different experts (representing research, policy and practice) and end-users. End-users of the Canteen Scan are experienced school canteen advisors, representatives of caterers (who provide the foods and designs of the canteens in several schools) and canteen managers/employees. Both qualitative and quantitative methods were used to provide complementary information and to improve the rigour of the study [50]. After each step, research results were discussed in the project team and the Canteen Scan was further improved.

In the Netherlands, most students bring their own food and drinks from home and buy food or drinks at school only as complementary foods (snacks and drinks). School canteens can consist of a point-of-sale display (where people ask for, or take, a product from a display/cooler/shelf and pay at the cash register), and/or vending machines for food/drinks. The school canteen can be run by an external catering company, the school itself, or by a combination of these two.

### Study procedure

#### Development of a paper draft of the Canteen Scan

##### Creating the draft tool

To operationalise the four constructs of the guidelines (basic conditions, availability on display and in vending machines, and accessibility), the project team generated a proposition for the methods and measurable items, based on earlier experience, scientific literature, and consultation with experts in nudging and social marketing. The project team consisted of seven multidisciplinary researchers in the fields of childhood obesity, nutrition, prevention and public health, nutritional professionals, and a school canteen advisor of the Netherlands Nutrition Centre. Discussed were: possibilities to make use of an existing database to classify products into healthier/less healthy products according to the current Dutch nutritional guidelines, different methods to assess the quantity of products [52, 53], and items to assess the accessibility of products using several nudging strategies.

##### Expert meeting

A first concept of the Canteen Scan was discussed with experts to reach consensus about the proposed methods, items and response options. Whilst ensuring the scientific evidence, the practical feasibility was taken into account. The expert meeting was attended by 19 of 22 invited experts from research and policy on nudging, nutrition and health behaviour, and professionals representing school, sport and worksite organisations/caterers. Prior to the meeting, attendees received the draft tool by email and were invited to add additional ideas to be discussed. The draft tool consisted of two parts: one part with a proposal to quantify food products and another with proposed items to assess accessibility. An external chairperson directed and structured the meeting that was audio-recorded and minuted. NW reviewed and summarized the results and this was checked by EV and CR. All attendees received the consensus document of the meeting and were asked to check the content.

##### Interviews and expert meetings with canteen managers/caterers and canteen advisors

To acquire feedback from end-users about the relevance, comprehensiveness and feasibility of the developed methods, items and response options, six semi-structured interviews and two expert meetings were held. The interviews were semi-structured in that specific questions of interest were posed but allowed the trained interviewer to probe questions if answers needed more explanation.

The interviews were conducted with two canteen managers and four representatives of caterers, representing different school canteens with diverse education levels throughout the Netherlands. During these semi-structured interviews (each lasting ±120 min) the Canteen Scan was filled out for the respective canteen. After completion, the relevance, comprehensiveness and feasibility of each proposed measurement method to assess availability and the criteria for accessibility were assessed with structured questions [48–50]. Examples of questions are: “*Is it possible to classify the offered products in the right food group?”; “What is your opinion about and which barriers/facilitators do you expect regarding selling fruit at the check-out counter*?” Furthermore, participants could add extra items they considered important. The feedback on each element of the Canteen Scan was sorted, reviewed and summarized by NW and checked by EV and CR.

During two expert meetings (with six and four school canteen advisors, respectively) from the Netherlands Nutrition Centre, the proposed methods and items were rated on feasibility (yes, maybe, no), barriers/facilitators were discussed and any suggestions for adaptions were addressed. The ratings on feasibility were counted and a summary of discussion points per Canteen Scan element was made. Afterwards, all attendees received and approved the conclusions that emerged. The results of the interviews and expert meeting were discussed in the project team and used to improve the tool.

Based on the three steps (1a, 1b, and 1c), a paper version of the Canteen scan was developed.

#### Assessing content validity of the paper draft of the Canteen Scan

It is important to assess content validity to be able to review whether users understand the questions as intended. To gain insight into the content validity, we assessed the concepts relevance, comprehensibility and comprehensiveness [48–50]. The paper draft of the Canteen Scan was assessed by four different end-users (canteen managers and representatives of caterers) in four schools with a medium size canteen. Schools differed in canteen operator (*n* = 2 by the school itself, *n* = 2 by a caterer) and expected healthfulness of the canteen (*n* = 2 healthier canteen, *n* = 2 not healthy). End-users were instructed to conduct the Canteen Scan in their canteen, which included two options to quantify the available products. First counting the numbers of products and second counting the rows per product (called ‘facings’). Subsequently, a structured interview was performed to review the content validity by the concepts relevance (does the instrument contain only relevant aspects?), comprehensibility (are all aspects understood as intended, and are the response options appropriate?), and comprehensiveness (are no important aspects missing?). In addition, feasibility and recommendations were assessed [48–50]. Each concept was questioned per construct of the Canteen Scan. E.g. *“Which method, counting or facings, represents the offer on display the best?”* and *“Is it feasible to select products you see first while moving along the route through the canteen?”*). At least, general open questions were stated, e.g. *“What is your opinion about the amount of time needed to fill-out the Canteen Scan?”* In addition to the structured questions, the trained interviewer (NW) was allowed to probe questions if answers needed more explanation. NW sorted, reviewed and extracted the results, and this was checked by EV and CR. The summarized findings were discussed in the project team and used to further refine the Canteen Scan.

#### Pilot testing the online version of the Canteen Scan

The refined paper version of the Canteen Scan was translated into an online tool which was pilot tested for its usability among four end-users from four different school canteens, which differed in canteen operator and expected healthfulness of the canteen. Pilot testing improves the adaptation of the tool by practice. It reveals missing items, interpretation problems and gives insight in how long it takes to fill out the tool [49]. End-users were invited to fill out the online Canteen Scan using an iPad in their canteen. Meanwhile, respondents were asked to think out loud as they filled in the Canteen Scan. This cognitive interview technique ‘think aloud’ was used to understand respondent’s comprehensibility and to reveal areas for improvements [50, 54]. Although this method is time-consuming, subjective, and its validity questionable, in combination with other methods, it can support the development of new tools [54]. In addition, the researcher asked questions if their thoughts were not clear. Thereafter, the usability of the online Canteen Scan was assessed by the concepts comprehensibility, user-friendliness (i.e. easy to understand), feasibility (i.e. practically applicable), time-investment and overall satisfaction [48–50]. Questions (answered on a 5-point Likert scale, ranging from 1 not feasible at all, to 5 very feasible) were asked to assess comprehensibility, user-friendliness and feasibility, structured within the five Canteen Scan elements; basic conditions (*n* = 2), availability on display and vending machines (*n* = 17), accessibility (*n* = 28), and result and feedback (*n* = 8), together with an overall opinion (*n* = 3). In addition, questions were asked with respect to the investment of time (*n* = 3), e.g. “The amount of time required to fill out the Canteen Scan was worth it” (5-point Likert scale: 1 totally disagree to 5 totally agree); the actual amount of time it took to fill out the Canteen Scan (minutes); and overall satisfaction (*n* = 1) (“In general, how satisfied are you with the Canteen Scan”, 5-point Likert scale: 1. very unsatisfied to 5. very satisfied). Mean scores were calculated and the “think aloud” results were summarized per element of the Canteen Scan by NW, and checked by EV and CR. These results were discussed in the project team to improve the tool.

## Results

Measurement methods, items and response options belonging to the four constructs (basic conditions, availability on display and in vending machines, and accessibility) were proposed, evaluated and refined in collaboration with experts, end-users and the project team during several rounds. The proposed items and main revisions during development are shown in Table 1.

**Table 1:** Proposition and revisions per step and per construct of the Canteen Scan

Element	Step 1: Development (expert meeting *n* = 19; interviews *n* = 6; second expert meeting *n* = 10)	Step 2: Content validity of paper scan (*n* = 4)	Step 3: Pilot testing the online scan	
			*Translation into online scan*	*Pilot testing the online scan (n = 4)*
Basic conditions	- 2 automatically assessed questions - 2 multiple choice questions		- 2 multiple choice questions were split into two different sets of questions.	- Improve formulation of the questions
Availability of food and drinks *- method to classify products*	Link the tool with the LEDA^a^	- Link with LEDA was evaluated positive		- Optimise the database - Buttons were difficult to find - Added the option to adapt entered composite products.
Availability of food and drinks *- method to assess quantity*	- Measuring relative shelf space - Combination of counting facings and product numbers on displays, racks, coolers - Counting facings in vending machines	- A product list with common products was suggested, to reduce time to enter a product - Counting facings on displays (racks, shelf) was infeasible. In coolers facings was feasible - Counting facings in vending machines was feasible	- Two separate elements were created for “food and drink on display” and “food and drinks in vending machines” to increase clarity. - The combination of facings and numbers in displays was technically infeasible, so all products need to be counted	- Difficult to fill in the Canteen Scan during opening hours as the assortment changes. - Numbers of product above 30 go in steps of 5.
Accessibility criteria	- Experts suggested to add “not applicable” to the response options - Reformulations were suggested - 3 Price items were proposed, but reduced to 1 on advice of experts, because infeasible/too costly for practice - 2 Items about prominent placing were split into three to improve comprehensiveness - A portion size item was suggested and rejected - Finally, 10 items were proposed	- Item for attractive presentation of fruit and vegetables was added [54] - 11 Items remained	- Three items for prominent placing were reduced to one item, to ensure equal contribution of placing regarding all accessibility items - 9 Items remained	- Add examples in text or picture, of the criteria - Questions to assess whether products are placed on the most eye-catching spot is reformulated
Results and feedback	- A score per construct and an awarded level - A general advice and a specific advice with all entered and classification of products - Portion size and pricing were added to the advice			- Improved the advises of the accessibility criteria

^a^LEDA = An existing database with most of the Dutch sold food/drink products, including their nutritional value

### Development of the paper draft of the Canteen Scan

During the first step, experts recommended to add a separate result and feedback section to make actions to improve the canteen very clear for people in practice. Experts agreed to count each number of products on display, and to count each facing for vending machines. Moreover, they recommended using the school canteen as priority setting during the development, due to the differences between the school, sport and worksites settings. Worksite cafeterias and sports canteens differ with respect to the products offered and physical size, compared to school canteens.

Pricing (e.g. offering healthier option at a lower price compared to less healthy options) and offering different portion sizes are highly potent strategies to stimulate healthy eating [55–57]. However, during the expert meeting (step 1b) schools and caterers reported these to be infeasible since the buying-in costs are higher for healthier options. Therefore, instead of adding this as an item, these strategies were included as a suggestion to improve the healthiness of the canteen in the feedback element.

### Assessing the content validity of the paper draft of the Canteen Scan

The second step showed a positive evaluation of the approach to count numbers of products on display and to count product facings in vending machines. Evaluation in four schools showed that the database of Dutch food and drink products (LEDA) is able to classify the entered products in the correct product group. 90% of the offered products on display could be classified into the correct product group, and for 96% in vending machines respectively. However, it was suggested to add a list with common products to reduce the time required to complete the scan. Regarding accessibility one item was added to stimulate attractive placement of fruit and vegetables.

### Pilot testing the online version of the Canteen Scan

During the translation of the paper draft into the online tool, it became clear that it was necessary to split the construct of availability into two sections: availability on display, and availability in vending machines. The pilot test with four canteen managers/representatives of caterers yielded an average score on the usability concepts comprehensibility, user-friendliness, feasibility, time investment and satisfaction of 3.4 to 4.6 (range 1–5, 5 represented very feasible) (Table 2). This indicates that on average all elements of the scan were evaluated positive (mean scores ≥4.0, range 3–5), except for time investment (mean score 3.4, range 2–5). Filling out the Canteen Scan took on average 127.5 (range 105–165) min. The accompanying thinking-aloud method revealed that the tool could be improved by adding more detailed instructions, optimising the database, reducing the completion time and making minor technical adjustments (e.g. position of buttons).

**Table 2:** Results of the pilot tests, per element of the Canteen Scan

Concept	Basic conditions^b^	Availability^c^	Accessibility^d^	Result and feedback^e^	Overall opinion^f^
	Mean (range)	Mean (range)	Mean (range)	Mean (range)	Mean (range)
Comprehensibility^a^	4.0 (3–5)	4.1 (2–5)	4.0 (1–5)	4.2 (2–5)	4.0 (4–4)
User-friendliness^a^	4.3 (4–5)	4.5 (2–5)	4.3 (2–5)	4.5 (4–5)	4.3 (4–5)
Feasibility^a^		4.6 (1–5)	4.3 (3–5)	4.0 (4–5)	4.0 (4–4)
Time investment^a^					3.4 (2–5)
Satisfaction^a^					4.0 (3–5)

^a^All measured on a 5-point Likert scale from negative to positive (e.g. very incomprehensible to very comprehensible)

^b^Basic conditions were measured with 1 comprehensibility and 1 user-friendliness question

^c^Availability was measured with 7 comprehensibility, 7 user-friendliness and 3 feasibility questions

^d^Accessibility was measured with 12 comprehensibility, 9 user-friendliness and 7 feasibility questions

^e^Results and feedback was measured with 4 comprehensibility, 1 user-friendliness and 3 feasibility questions

^f^Overall opinions were measured with 1 question for each concept, except for time investment which was measured with 3 questions

### Description of the Canteen Scan

**Fig. 2:**
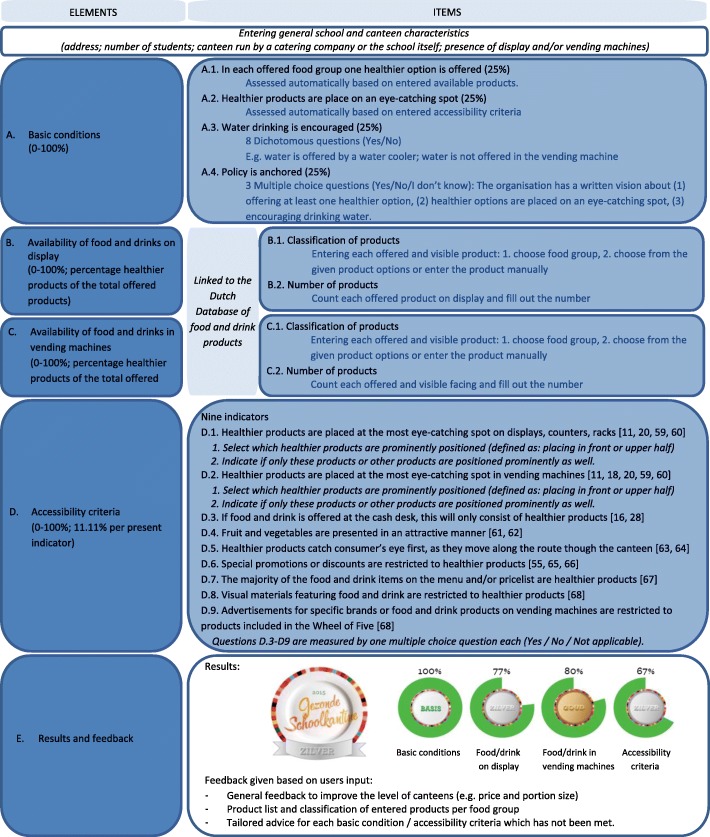
Description of the Canteen Scan

These three steps resulted in the online Canteen Scan consisting of five elements: A) basic conditions, B) availability of food and drinks on display, C) availability of food and drinks in vending machines, D) accessibility criteria, and E) results and feedback (Fig. 2). All elements of the Canteen Scan include information buttons with detailed explanations and examples. The input can be copied and adapted to monitor changes over time.

#### Element A: Basic conditions

The first element contains four basic conditions for a healthier canteen. Each condition can be scored as being present (25%) or not (0%), summed together to 100% (Fig. 2). Two of the four basic conditions (A1. *“In each food group one healthier option is offered”* and A2. *“Healthier products are placed on an eye-catching spot”*) are based on the information filled in under the availability and accessibility elements. The other two conditions (A3. *“Encourage water drinking”* and A4. *“Availability of policy”*) were assessed using 8 dichotomous and 3 multiple choice questions, respectively.

#### Elements B and C: Availability of food and drinks

All available products can be entered in the scan by selecting the corresponding food group (11 food groups in total, e.g. vegetables, main course salads, fruits, sandwiches, bread, dairy), and selecting (in case of the most frequently sold products) or entering (typewriting) the product. Products are then automatically classified as a healthier or less healthy products, based on the linked Dutch database LEDA [58]. If products are not present in the database, the product and their calorie content can be added manually. Composite products (sandwiches/salads) can be added manually by entering the individual constituents (e.g. of a “whole-wheat sandwich cheese” the kind and amount of bread, margarine, cheese, lettuce and tomatoes can be added). A composite product is categorized as a healthier product if the main ingredient (bread, salad) is a healthier product and the sandwich toppings are less than 30 g, and sauces are limited to one eating spoon. The amount of each product (in case of displays/racks) or the number of facings of each product (in vending machines) has to be entered, on which the proportion of healthier products to the total number of products (or facings) is calculated.

#### Element D: Accessibility criteria

Accessibility is assessed by nine items that are scored yes/no/not applicable (Fig. 2). These items assess effective strategies to increase healthier choices through either product placement (5 items) or promotion (4 items) [11, 16, 18, 20, 28, 55, 59–68]. The score for accessibility is calculated as the percentage of fulfilled criteria (0–100%) relative to all applicable criteria.

#### Element E: Results and feedback

The result section of the Canteen Scan consists of four separate percentages for each of the above-mentioned elements. All basic conditions need to be present and the lowest percentage among the scores for availability and accessibility determines the awarded level of either bronze, silver or gold.

In addition to the awarded level, both general and tailored feedback to improve the canteen is provided. For example, general advice regarding portion sizes and pricing is given, as well as an overview of all available products and their classification. A tailored advice is given for each basic condition or accessibility criteria which has not been met (e.g. “Place fruit and vegetable next to the cash desk and place less healthier products at another less visible place”).

## Discussion

The present study translated the Dutch Guidelines for Healthier Canteens into an online tool called the ‘Canteen Scan’ in a 3-step iterative process. The Canteen Scan provides insight into the level of compliance with the guidelines, and offers feedback with directions for improvement. The tool was developed for and with various users, e.g. (school) canteen advisors/managers/employees and caterers, as well as involving stakeholders representing science and policy. Pilot tests revealed that stakeholders evaluated the tool positive on its usability, with positive evaluations on the concepts comprehensibility, user-friendliness, feasibility and satisfaction.

Besides the Netherlands, other countries have developed guidelines or policies and accompanying tools to stimulate healthy eating behaviour in public settings [15, 39, 42, 44]. Unfortunately, none of the available tools were suitable to monitor Dutch school canteens due to the differences in goals, criteria and the definitions used. The Canteen Scan was specifically developed to evaluate compliance with the Dutch guidelines for canteens, according to Dutch nutritional guidelines, suitable for the products sold in Dutch school canteens and with the recommended definition (by stakeholders) of accessibility. However, the process of development and the content of the tool can be valuable to others developing a similar tool for their canteens.

To our knowledge, the Canteen Scan is the first online tool to translate policy for public food settings into a tool that combines assessments of the healthiness of products, the proportion of healthier products available in a canteen, and criteria for accessibility. In the present study, end-users evaluated the different elements of the Canteen Scan as positive on comprehensibility user-friendliness and feasibility. The combination of concepts (availability and accessibility) concurs with the recommendations of earlier tools developed to measure the consumer food environment [15, 44]. The tool can be used by a diversity of stakeholders: school managers, canteen employees, caterers, school canteen advisors and policy makers. In accordance with recommendations, the Canteen Scan combines the functions providing insight into the current level of compliance with guidelines, monitoring changes over time, and providing tailored feedback to improve the healthiness of the canteen [19, 35, 42, 44]. Moreover, since the adjustments with regard to accessibility/availability are immediately apparent in the result section of the tool, this may stimulate caterers and canteen managers to make changes. As the Canteen Scan is administered online, stakeholders could easily use the scan to monitor changes in healthiness over time. Another strength of the Canteen Scan is that it is linked with the Dutch database that automatically classifies commonly sold food/drink products according to the current Dutch nutritional guidelines, based on the nutritional composition of products. The fact that users themselves do not have to classify products increases the usability of the tool [42, 69]. Moreover, this link allows to automatically include updates of the nutritional guidelines in the Canteen Scan. On national level, the (anonymized) online data might be used to monitor how many organizations implement and comply with the Guidelines for Healthier Canteens, although first more insight should be gained in the reliability and validity of the tool. The monitoring of implementation and compliance to guidelines is recommended to be able to evaluate the (un)intended effects of stated policy and to improve policy in the future [42]. Taking all this together, the Canteen Scan appears to be a useful tool for practice.

A limitation of the tool and a possible barrier for implementation [70] is that the use of the Canteen Scan was perceived to be time-consuming. Other comparable tools assess a more limited range of food groups, which can decrease entry time [45, 69]. However, we chose to assess all food groups and products in order to obtain more comprehensive insight into the assortment, to be able to observe changes in the assortment, and to provide insight to users on whether replacement of certain foods actually improves their score. In addition, pilot tests showed that the investment of time was worthwhile and improvements in the database can decrease the amount of time required. Moreover, the second and subsequent uses of the scan will be less time-consuming because a previously entered scan can be copied and simply adapted.

Another limitation is that some of the items used to score accessibility are difficult to quantify and, therefore, to measure. For example, the item *“healthier products are placed at an eye-catching spot”* is liable to bias because “eye-catching spot” can be interpreted in different ways. Therefore, to reduce possible bias, additional explanation by text and pictures to each item might be a solution.

To increase usability in practice, collaboration of science and practice is recommended for the development of such a tool [49, 50]. However, one of the challenges was to balance the needs and wishes from practice and the scientific evidence and to be able to align this with the technical possibilities. Consequently, certain compromises had to be made. For example, although price- and portions sizes strategies are effective [55–57] they were not included as accessibility item in the tool. By practice, this was considered not yet feasible since the buying-in costs are higher for healthier options. As solution, these strategies were added as a suggestion in the general feedback. The limited number of participating stakeholders that were consulted could have influenced the results. However, we included a wide range of stakeholders (researchers, school canteen advisors, professionals representing caterers and schools) to receive a broad range of information.

The development of the Canteen Scan is a continuous process and the tool will be adapted based on input from experts and end-users. This study showed the first refinements of the measurement methods and items of the Canteen Scan based on the input of the experts and end-users. In a follow-up (quantitative) study, the criterion validity and reliability of the Canteen Scan will be investigated in a larger sample, which should lead to further improvements.

The Guidelines for Healthier Canteens are applicable in school/sports canteens and worksite cafeterias. During the expert meeting in the first step of the development of the Canteen Scan, experts advised us to focus on school canteens. Based on the noticed differences between the settings, e.g. different products, more meals on offer, and a different organisational structure (i.e. more volunteers in sports canteens). However, currently the Canteen Scan is already used in sport and worksite canteens. Based on these experiences, future refinements will be made to increase the Canteen Scan’s usability also in other settings than the school setting, such as sports canteens and worksite cafeterias.

In the future, the Canteen Scan could be combined with measurements of the broader environment, e.g. in a daily life environment (such as home, neighbourhood or shops passed on the way home). In addition, investigating the relation between the objective consumer environment (measured with the Canteen Scan) and individual purchase and eating behaviour, health outcomes and perceptions of the environment (e.g. how important price is for the consumer) might increase knowledge on the food environment and the relation with individual behaviour and health [31, 71].

## Conclusion

The Canteen Scan was developed in collaboration with experts, end-users and researchers, thereby balancing scientific and practical considerations. The tool will provide stakeholders insight into the level of compliance with the Dutch Guidelines for Healthier Canteens and will offer instant tailored feedback to support adjustments towards healthier canteens. As well, pending confirmation of the reliability and validity of the tool, the tool may be useful for canteen managers to monitor improvements in the healthiness of their canteen or for monitoring implementation of the guidelines on a national level. Pilot tests showed this tool to be comprehensive, user-friendly and feasible in daily practice. Further research is needed to elucidate to what extent the tool actually supports schools and caterers to create and sustain healthy canteens.

## Abbreviation

LEDA: An existing Dutch database with most of the Dutch sold food/drink products, including their nutritional value
